# Effect of Aluminium Substitution on Physical Adsorption of Chloride and Sulphate Ions in Cement-Based Materials

**DOI:** 10.3390/ma16176029

**Published:** 2023-09-01

**Authors:** Guangtai Zhang, Maoquan Li, Zheyu Zhu

**Affiliations:** 1 Institute of Civil Engineering, Xinjiang University, Urumqi 210094, China; wig59139qqecm@163.com; 2 Xinjiang Key Laboratory of Building Structure and Earthquake Resistance, Urumqi 830017, China; 3 School of Materials Science and Engineering, Yancheng Institute of Technology, Yancheng 224051, China; 17712937215@163.com

**Keywords:** chloride ion, sulphate ion, molecular dynamics, scanning electron microscope, physical adsorption, aluminium substitution, transition interface

## Abstract

When aluminium-rich phase minerals are added to Portland cement, Al atoms will enter the C-S-H and Al, then a substitution reaction will occur, forming a hydrated silica-calcium aluminate (C-A-S-H), which changes the molecular structure of the cement material. Due to limitations in experimental methods, the research on the bonding effect between corroded ions and Al-substituted structures is still unclear. Here, the mechanism of an Al substitution reaction affecting the adsorption of chloride and sulphate ions was studied using simulation. The C-A-S-H model of aluminium random substitution was built, evaluating the binding effects among the C-A-S-H, and sulphate and chloride ions. The results demonstrated that the C-A-S-H structure generated by the Al substitution reaction increased the physical adsorption capacity of the chloride and sulphate ions. The adsorption capacity of the sulphate ions was 13.26% higher than that before the Al substitution, and the adsorption capacity of chloride ions was 21.32% higher than that before the Al substitution. The addition of high aluminium phase minerals caused the interfacial flocculants C-A-S-H and C-S-H to connect and intertwine in the the interface transition zone (ITZ) structure. The addition of high-alumina phase minerals improves the microstructure of concrete hydration products, improving the physical and mechanical properties and durability of concrete. After the addition of 20% lithium slag, the sulphate ion erosion content and the chloride ion erosion content of the concrete decreased by 13.65% and 15.72%, respectively. This paper provides a deeper understanding of the effect of high-alumina phase admixtures on concrete at the micro-scale.

## 1. Introduction

Concrete is an extensively employed construction material, with an estimated annual consumption of roughly 6 billion tonnes in the industry. To achieve economic and environmental objectives, cementitious materials and mineral admixtures are generally added to concrete, enabling the full utilization of industrial waste and a reduction in carbon emissions. Endurance, a crucial determinant of concrete’s long-term performance, ensures the sustained use of concrete structures. However, service conditions greatly affect the durability of concrete structures, especially in high chloride-, sulphur-rich environments like oceans and saline land areas. After corrosion by chloride ions, the reinforcement’s service life decreases significantly because of concrete crystallization and ettringite chemical erosion caused by sulphate ion attacks. Chloride and sulphate ions, representing two different forms and penetrating the interior, contribute to the corrosion, in which the first is free Cl^−^ and SO_4_^2−^ immersed in the concrete pore solution. The second form is Cl^−^ and SO_4_^2−^ bound with cement hydration products, which includes chemical bonding (solidification) and physical adsorption. Both forms of Cl^−^ and SO_4_^2−^ are in equilibrium with each other. However, the alteration of environmental conditions gives rise to a change in free Cl^−^ and SO_4_^2−^ concentration, combined Cl^−^ and SO_4_^2−^ content, and even the desorption of physically adsorbed Cl^−^ and SO_4_^2−^. This process has the potential to drive physically adsorbed Cl^−^ and SO_4_^2−^ to penetrate deep into the concrete’s interior. Therefore, exploring physical desorption factors contributes to the study of corrosion resistance when subjected to chloride-rich and sulphur-rich environments. Cement-based materials can enact the physical adsorption of harmful ions, primarily governed by the van der Waals force and electrostatic force of C-S-H gel [1,2].

Calcium silicate hydrate (C-S-H) is a crucial constituent of cement, but the presence of aluminium doping in this structure is a commonly accepted phenomenon, as noted by Merlino [3]. Nowadays, mineral admixtures are widely utilized to partially replace cement in concrete production to achieve the goal of a low-carbon economy. With admixtures having a high aluminium content, more Al is incorporated into the C-S-H structure, leading to Si replacement at the bridge site and creating a novel aluminosilicate spatial structure product-C-A-S-H. This mechanism further promotes the formation of hydrated silicon calcium aluminates (C-A-S-H). It is reported that the incorporation of Al induces the formation of three-dimensional lattice architectures in the silicon-aluminium oxide chains, which significantly increases their molecular chain lengths and polymerisation degrees [4]. Substituted skeletal structures are more stable than the C-S-H framework, improving the mechanical properties and corrosion resistance of concrete. L’Hôpita [5] carried out experiments to demonstrate that Al doping enhances the adsorption capacity of alkali metal cations, such as Na^+^ and K^+^ within the structure.

The addition of high alumina minerals can significantly impact the physical and chemical properties of cement materials. He [6] have determined that high alumina mineral admixtures can alter the microstructure of cement. Specifically, the morphology of the C-S-H gel changes from irregular flaky particles to spherical particles that are densely packed. Through extensive experimental research, scholars [7,8] have found that the inclusion of lithium slag, a high alumina mineral admixture, results in a decrease in the permeability of chloride ions in concrete. This reduction in permeability is attributed to the active mineral admixture reacting with cement hydration products after the addition of lithium slag, thus improving the compactness of concrete. However, it remains unclear how the molecular spatial structure changes impact ion permeation and bonding effects between corrosive ions and silica aluminate, as experimental limitations prevent direct observation of such effects. Therefore, simulation studies are required to investigate the effects of Al substitution reactions following the addition of lithium slag on the proportion of C-A-S-H structure in cement, as well as the bonding and adsorption amount of physical adsorption of chloride and sulphate ions.

In this study, molecular dynamic simulations were employed to establish an adsorption model for Cl^−^ and SO_4_^2−^ on the surface of C-S-H and C-A-S-H following the substitution of Al. A range of dynamic analysis methods were performed to determine the coordination number, adsorption site, adsorption amount, and binding effects of these ions in order to assess the adsorption amount of Cl^−^ and SO_4_^2−^ by C-A-S-H and C-S-H. By examining the interfacial transition zone of concrete, following the inclusion of lithium slag, the effect of introducing highly active aluminium phase compound mineral admixtures on the durability of concrete materials was explained from a microscopic perspective. SEM and XRD techniques were utilized to facilitate this examination.

## 2. Experiment

### 2.1. Materials

(1) Cement: P·O42.5 ordinary Portland cement from Xinjiang Midong Tianshan Cement Co., LTD. (Urumqi, China) Coarse aggregate: fine pebbles with a particle size of 5–25 mm; fine aggregate: medium sand with less mud content after sifting and washing; water: mixing water using Urumqi tap water; and lithium slag, see Table 1 for chemical composition.

Sodium sulphate: anhydrous sodium sulphate; Sodium chloride: Anhydrous sodium chloride.

(2) Concrete mix ratio

The concrete uses C40 strength concrete. After our team’s in-depth research, the replacement rate of lithium slag can reach 20% of the cement mass, see Table 2 for details.

### 2.2. Test Scheme

C40 strength grade lithium slag concrete specimens were prepared in which the volume replacement rates of lithium slag were 0%, 10%, and 20%. Three samples with different replacement rates were prepared in the erosion solution of 25 concentration combinations, a total of two hundred and twenty five 150 × 150 × 150 mm cube test blocks were strictly sealed with four sides of epoxy resin, and they were then put into the erosion solution with different concentration gradients, according to the proportions of 0%, 2%, 4%, 6%, and 8% to ensure their quality. After a long soaking time of 150 days, the samples were taken out and the surface of each sample was obtained by drilling holes for powder extraction. The drilling treatment was carried out at different depths, and then the powder was put into appropriate distilled water to form a suspension. The concentration of the chloride and sulphate ions dissolved from the top layer of concrete was then measured and converted to give the content of chloride and sulphate ions penetrating each sample.

The specific test steps are as follows:

(1) The size of the concrete test block is 150 × 150 × 150 mm. To create one-dimensional erosion conditions, four sides of the specimen were coated with epoxy resin to form a hydrophobic protective layer on all sides.
(2) A mixture of NaCl and Na_2_SO_4_ was used to prepare the erosion solvent, in which the chloride and sulphate contents were arranged in the order 0%, 2%, 4%, 6%, and 8%, with a total of 25 different ratios (including the control group).
(3) Drill holes for extracting powder.

After 150 days of erosion, the sample is removed from the water bath and allowed to dry naturally. Then 5 mm, 10 mm, 15 mm, 20 mm, 25 mm, and 30 mm holes are drilled along the eroded surface and these powder samples are packed in an airtight bag for later use.

(4) Determination of chloride and sulphate ion content

**Figure 1:**
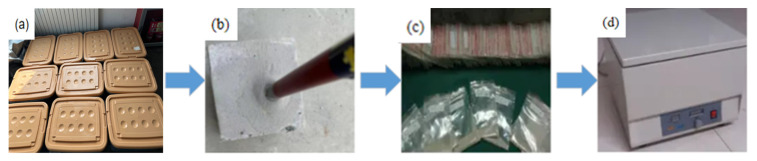
Schematic diagram of experimental steps (**a**) gradient solution erosion, (**b**) drilling holes on concrete surfaces, (**c**) powder sample, (**d**) Chloride and sulphate ion content testing.

The experimental steps are shown in Figure 1. First, the powder sample is dissolved in a container with distilled water, the container is placed on the surface of the oscillator for 30 min of oscillation, maintaining the safety time of 24 h after filtering out the supernatant as the liquid to be measured, then, according to the standard operation, a chloride ion detector and sulphate ion detector is used detect the solution. Finally, the content of the chloride and sulphate ions in the powder is calculated by converting the concentration of chloride and sulphate ions in the liquid to be tested.

Determination of the chloride ion content: 1. Pour 4 g of powder into a container with a capacity of 40 g of distilled water, place the container on the surface of the oscillator, oscillate for 30 min, and then maintain a standing time of 24 h. 2. Calibrate the electrodes of the DY-2501A chloride ion meter. The calibration procedure is as follows: insert the electrode into the marked electrode, and the value of the control SLP ranges from 90% to 110% in the calibration solution of the different concentrations. 3. Insert the calibration electrode into the bottle to be tested; after an accurate calibration, ensure that the electrode potential is stable, and then the mass concentration of chloride ions can be accurately measured.

Determination of sulphate ion content: 1. Weigh ag powder accurately, pour it into a container filled with 60 mL of distilled water, place it on a shaking table for 30 min, and then let it stand for a period of time; filter out 50 mL of supernatant as the liquid to be measured, and finally pour it into a No. 1 beaker. 2. Drop ammonia into beakers 0 and 1 to turn it into a light lemon colour, then continue to drop in 2 drops until the solution is completely set, and finally adjust it to 50 mL. 3. Slowly pass the liquid through the dry quantitative filter paper; discard the first 5 mL of filtered filtrate, collect the remaining filtrate in a colorimetric dish; then, just like the sulphate tester, first clear the sample tank to read the volume concentration of the sulphate ions in the liquid to be measured.

### 2.3. Simulation Methods and Parameters

**Figure 2:**
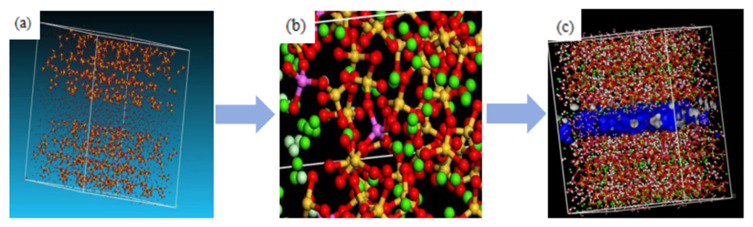
Model establishment process (**a**) establishment of C-S-H crystal mode, (**b**) selection of Q^2p2b^ and Q sites replaced by Al, (**c**) replacement of C-A-S-H model.

A review of the various calcium silicate hydrate models over the past half century shows that although there are differences between the models, they are very close in essence. Many models are based on the structural unit tobermorite, of which tobermorite is an important component [8,9,10,11,12,13], but it is also an important basis for the development of molecular dynamics models. Through FTIR experiments and molecular dynamics simulations, Zhao [9] et al. found that the 11 Å tobermorite structure is the best basis for the formation of the C-S-H phase in the initial stage of cement hydration. This article constructs a preliminary C-S-H model based on the 11 Å tobermorite crystal, as shown in Figure 2a.

Due to the presence of a certain amount of CA_3_ and C_4_AF components in cement, and the presence of a large amount of aluminium phase components in the mixed lithium slag, all these will lead to the occurrence of Al substitution reactions and the formation of calcium aluminate silicate hydrate (C-A-S-H), which will affect the structure and mechanical properties of the cement, further affecting the diffusion of ions in C-A-S-H, and then affecting its corrosion resistance. According to the literature [14], aldoping mainly occurs at the Q site^2b^, but in the presence of sodium and potassium ions, substitution can also occur at the Q site^2p^. Myers [15] developed the CTSM model, which targets the branching structure (Q) in the C-A-S-H gel. Based on this, this paper replaces Al atoms at specific sites to match the Al doping reaction. Firstly, the removal chain is randomly selected and then the Si at Q^2p^ and Q sites^2b^ in the silicon chain are replaced. After replacing, the pink particle in Figure 2b is an Al atom. The deleted bridged silicon site is randomly selected and replaced with an aluminium atom at that site, forming an aluminium-silicon chain structure.

The maximum total aluminium/(Si + Al) ratio in the C-S-H of the sample is about 23%, with a typical value of about 21%. When building the model, the sites that meet the conditions for substitution are controlled, and the proportion of Al substitution in the final model is optimized, accounting for 18% of the total silicon atoms in the initial C-S-H model. The overall model of C-A-S-H after substitution is shown in Figure 2c.

**Figure 3:**
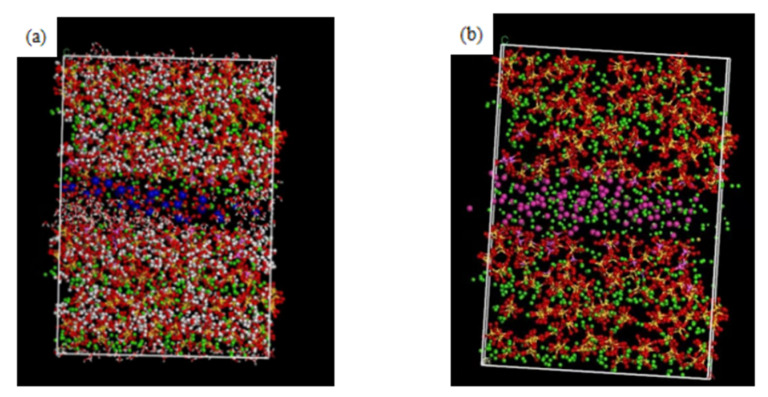
C-A-S-H Layered structure (**a**) 3.5%Na_2_SO_4_, (**b**) 3.5%NaCl.

Erosion ions are added to the model to simulate the 3.5% Na_2_SO_4_ solution used in the 3.5% NaCl solution, as shown in Figure 3a,b, and the corresponding concentration of Cl^−^ is 0.684 mol/L and SO_4_^2−^ was 0.280 mol/L. The interlayer distance in the model is set to 30 Å; the diffusion and adsorption of the ions into the interlayer of the C-A-S-H gel are simulated using the molecular dynamics method, and the physical adsorption of the eroded ions into the pores of the C-A-S-H gel is simulated using the COMPASS2 force field. The force field is based on ab initio calculations of atomic valence parameters and partial atomic charges. The van der Waals parameters are calculated by fitting the experimental data; the van der Waals interactions are computed using liquid molecular dynamics simulations [16], which can be used to model the physical adsorption problem in this study. The electrostatic interactions between molecules are calculated using the Ewald summation method, and the van der Waals forces between the molecules are calculated using the Leonard Johns interaction potential.

The simulation procedure is as follows: 1. The replaced C-A-S-H model is fixed, the geometry of the interlayer solution of the model is optimised and the initial configuration with the lowest energy is selected. 2. After the model is in equilibrium, dynamic relaxation is performed on the entire model. The temperature is set to 303 K and the NVT ensemble is selected. The total simulation time is 3000 ps, with a time step of 1 fs. The first 1000 ps simulated is divided into 100 ps, 400 ps, 700 ps, and 1000 ps, and the time interval of the last 2000 ps is set to 1000 ps. The configuration information is sampled every 100 fs to obtain the equilibrium dynamic trajectory of the atomic configurations. Through the radial distribution function (RDF), coordination number (CN), adsorption capacity, and other parameters, the influence of the C-A-S-H model on the physical adsorption of the eroded ions is analysed.

## 3. Results and Discussion

### 3.1. C-A-S-H Adsorption Sites

#### 3.1.1. Cl^−^ Adsorption by C-A-S-H

According to the thermodynamic theory and the literature [17], the physical adsorption behaviour of the interlayer solution occurs in C-S-H with a high specific surface area. The surface of C-S-H adsorbs Cl- in two ways, one by binding with SIOH and the other by binding with the SiOCa+ group formed on the surface of C-S-H, as shown in Formula (1).

(1) $$\begin{array}{l} \equiv {\mathrm{SiOCa}}^{+}{+\;\mathrm{Cl}}^{-}↔\equiv \mathrm{SiOCaCl}\\ \equiv \mathrm{SiOH}+{\mathrm{Cl}}^{-}↔\equiv {\mathrm{SiOHCl}}^{-} \end{array}$$

**Figure 4:**
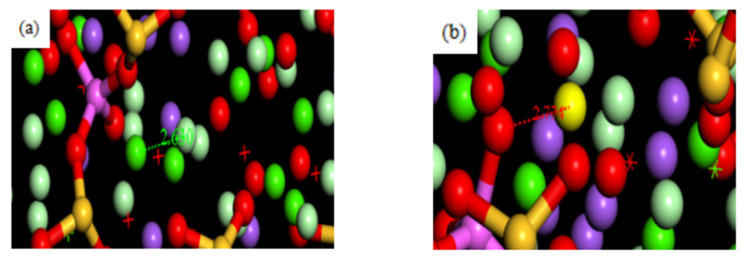
Binding form of the adsorption site on the surface of Cl^−^ and C-A-S-H (**a**) AlOCa^+^ and Cl^−^ key points Figure, (**b**) AlOH and Cl^−^ key points.

Figure 4a,b shows the bonding point between AlOCa^+^ and AlOH and Cl^−^ after substitution by Al. As the positive charge of Al is less than that of silicon, the interlayer Coulomb effect is also reduced, so the bond length of Al-O (1.62 Å) is slightly greater than that of Si-O (1.56 Å). Due to this substitution effect, the bonding of AlOCa^+^ with AlOH and Cl^−^ in the model will also change.

**Figure 5:**
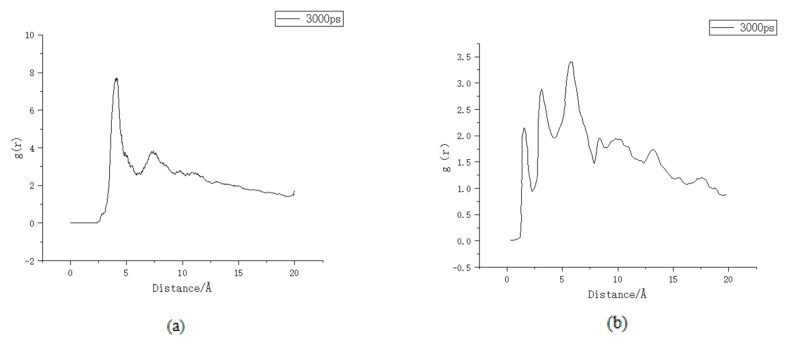
RDF image (**a**) AlOCa^+^-Cl^−^, (**b**) AlOH-Cl^−^ RDF.

Comparing both before and after the substitution, the bond length formed by the AlOCa^+^ group and Cl^−^ after substitution with Al was not much different from that formed by the SiOCa^+^ group and Cl^−^, while the bond length of the AlOH group and Cl^−^ was slightly larger. The RDF images of the AlOCa^+^ groups and AlOH and Cl^−^ are shown in Figure 5a,b.

The larger the value of g(r) in the RDF plot, the more ions are present around the adsorption site. As can be seen from the figure, the larger the value of g(r) in the plot of AlOCa^+^-Cl, the more Cl^−^ is adsorbed after the substitution, while the smaller the value of g(r) in the plot of AlOH-Cl indicates that the degree of adsorption is low. At the same time, the probability curve has no obvious peak value, so the molecular structure of AlOH is similar to that of SiOH, and the adsorption stability of AlOH for Cl^−^ is not strong. However, because the AlOCa^+^ group carries a positive charge, its adsorption is stable and the amount of Cl^−^ adsorbed is large.

According to Formula (1), the SiOCa group is electrically neutral and relatively stable after the adsorption of Cl^−^ before substitution, whereas the SiOH group is electronegative after adsorption of Cl^−^ and will repel Cl^−^ in solution, so it is unstable, and desorption occurs in solution. After substitution, although Al has less charge than Si, the local AlOH is electrically neutral due to the charge balance of the neighbouring atoms in the molecule. Therefore, the group formed with Cl^−^ after adsorption is still electronegative and the adsorption is also unstable.

According to the x-coordinate analysis of the RDF diagram of AlOCa^+^-Cl and AlOH-Cl, the peak of AlOCa^+^-Cl is about 3.10 Å, while that of AlOH-Cl is about 2.40 Å, indicating that the bond length of AlOCa-Cl and AlOH-Cl is about the same. When Al is replaced, the intensity of the first peak remains almost unchanged, while the intensity of the second peak increases, and the peak formed by the action of the van der Waals effect shifts to the right. This is because the AlOCa^+^-Cl ionic bonds formed by the physical adsorption are not stable, so they cannot be continuously confined to a fixed range by the Coulomb force of Ca^2+^. Desorption occurs with atomic motion. However, due to the large range of the long-range van der Waals effect, they will still dissociate on the surface region of the model after desorption. Compared to before the substitution, the overall positive charge of the model is reduced. In order to balance the structural charge of the model after substitution, the surface of the model will attract the free cations compensating in the solution. Laidler and Meiser [18] discovered that the negative charge on the surface of cement hydration products can attract Ca^2+^ and Na^+^ from alkaline pore solutions to form stern or adsorption layers. The presence of the stern layer causes the surface of the hydration products to tend towards a positive potential, attracting anions and diffusing them out of the stern layer to achieve potential neutralisation at the surface or edge of the stern layer. The adsorption capacity of the C-S-H gel for ions is highly dependent on the surface area of the C-S-H gel and the potential difference between the stern layer and the diffusion layer, also known as the zeta potential. The compensated cations improve the zeta potential of the model so that the long-range effect is enhanced, and the range of action is slightly increased, indicating that the peak of the van der Waals effect is shifted to the right.

#### 3.1.2. SO_4_^2−^ Adsorption by C-A-S-H

**Figure 6:**
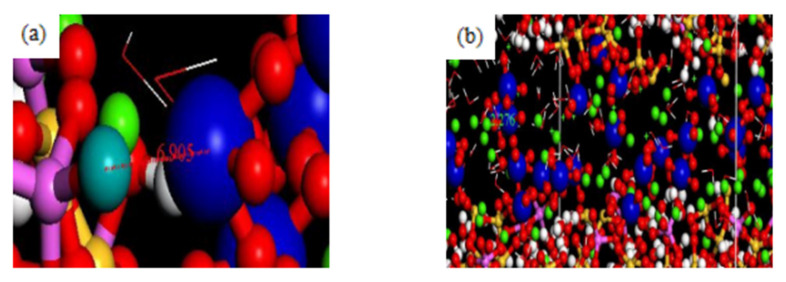
Binding form of adsorption sites on the surface of SO_4_^2−^ and C-A-S-H, (**a**) Enrichment of AlOH and SO_4_^2−^ unbonded, (**b**) Ca^2+^ on the surface of the model.

**Figure 7:**
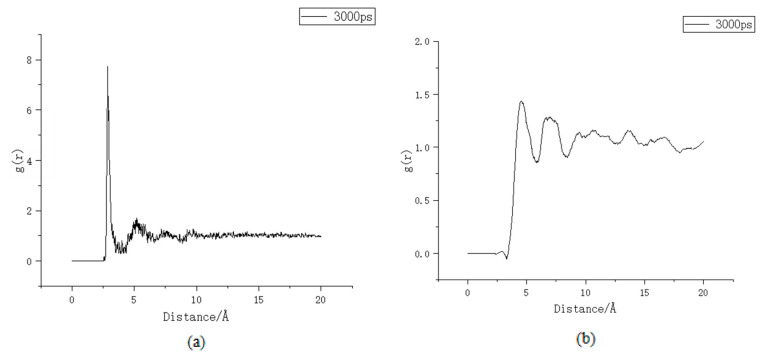
RDF (**a**) AlOCa^+^ and SO_4_^2−^, (**b**) AlOH and SO_4_^2−^.

If the erosive ion is SO_4_^2−^, the model also has the adsorption site for SO_4_^2−^. Figure 6a shows the adsorption sites of C-A-S-H and SO_4_^2−^. The substituted AlOH is still partially electrically neutral, it still cannot balance the positive charge carried by SO_4_^2−^, and AlOCa+ can still bind stably to SO_4_^2−^ due to its positive charge. From a global perspective, in order to compensate for the missing charge after the substitution of Al in the global model, some Ca^2+^ cations are attracted to the surface of the C-A-S-H skeleton, forming a double electrical layer structure. It can be seen from Figure 6b that a large number of Ca^2+^ ions are enriched around the model at the Al substitution site. Some of the Ca^2+^ is adsorbed onto the surface of the C-A-S-H model. At this time, the positive ion charge on the surface of the model is increased and a double electrical layer structure is formed to bind SO_4_^2−^ more effectively. Figure 7a,b shows the RDF diagram between AlOCa^+^ and AlOH and SO_4_^2−^.

It can be seen that AlOCa^+^ and SO_4_^2−^ RDF graph has a high peak value near 2.6 Å, which also indicates that AlOCa group can adsorb SO_4_^2−^ and is relatively stable. However, there is no obvious peak and regularity in the AlOH and SO_4_^2−^ RDF graph, which indicates that AlOH cannot have a significant adsorption effect on SO_4_^2−^.

### 3.2. C-A-S-H versus C-S-H Adsorption Capacity

Rao [19] et al. used the double electric layer (EDL) theory to explain the adsorption effect of C-S-H gel surface on chloride ions. According to the research of Kevin [20], because the surface layer of cement has a negative charge, it can react with Ca^2+^ and Na^+^ in an alkaline pore solution to form the stern layer. The existence of a stern layer will cause the surface layer of cement to be positively charged, thus promoting the diffusion of anions to the surface layer of the cement. To achieve a potential equilibrium in or around the stern layer. The adsorption capacity of the C-S-H gel depends on its surface area interacting with the stern and diffusion layers. The extent of this interaction can be measured using the zeta potential.

Elakneswaran [21] et al. found that the zeta potential value and positivity of cement slurry mainly depends on the Ca^2+^ concentration in the pore solution, which affects the physical adsorption of the chloride ions by cement-based materials. Usually, Ca^2+^ is saturated in the pore solution of cement-based materials and can combine with the silanol-based surface to form a positive potential C-S-H gel. Cement-based materials have a large number of silanol groups, and the existence of silanol groups is the main factor affecting the formation and performance of the electrical double layer. Due to the high surface potential of the C-S-H gel under the action of a surface potential, the chloride ions in the pore solution will be physically adsorbed onto the surface of the C-S-H gel.

The adsorption equilibrium equation is as follows:

SiOH + Ca^2+^ = SiOCa^+^ + H^+^

SiOH + Ca^2+^ + Cl^−^ = SiOCaCl + H^+^

SiOH + Ca^2+^ + SO_4_^2−^ = SiOCaSO_4_^−^ + H^+^

The adsorption of Ca^2+^ onto the surface of C-S-H increases the charge of the positive ions on the surface of C-S-H so that the double electrical layer binds more Cl; the tendency is for Ca^2+^ to bind more Cl^−^ than Na^+^. The charge characteristics of the C-S-H gel depend on its chemical composition, in particular the calcium-silicon ratio. When the calcium-silicon ratio is high, it will have a positive charge on its surface, and the silanol groups on the surface will combine with the Cl^−^ and OH^−^ anions in the pore solution to form a stable charged structure. If the calci-Si ratio of the C-S-H gel falls below 1.2, its surface will generate a negative charge [22] and K^+^, but chloride ions will remain in the pore solution.

**Figure 8:**
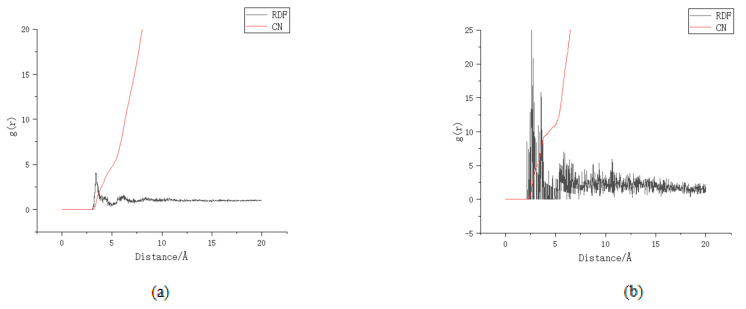
Coordination number (**a**) unsubstituted Si-Ca, (**b**) after substitution Al-Ca.

After the aluminium substitution, the calcium-silicon ratio also increases, and the ratio of interlayer free Ca^2+^ also increases, leading to an increase in the zeta potential of C-A-S-H; the change in potential will also lead to a change in the coordination number. After Al substitution, the charge of the whole model is no longer balanced due to the lack of a positive charge before substitution. In order to compensate for the missing positive charge, there will be a redistribution of free Ca^2+^ positions in the model. As a result, the physical adsorption of the chloride ions is affected, Figure 8a,b shows the coordination number before and after Al substitution.

According to the literature [23], before Al substitution, the coordination number of calcium ion is about 4, which is close to the fitting result of this model. However, the coordination number of substituted Ca^2+^ is about 7, which is similar to the coordination number result of calcium ion in the literature [24]. This indicates that after substitution, more Ca^2+^ ions are adsorbed by the C-A-S-H surface in the model system, resulting in the increase in stern potential. In order to balance the potential [25], the number of sulphate ions and chloride ions in the adsorption layer increased compared to that before the substitution. The literature [24] proposed Formula (2) to calculate the physical adsorption capacity of the adsorption sites using the isothermal adsorption method:

(2) $$\begin{array}{l} n^{a}=\frac{n_{ions}}{m_{C-S-H}}\\ n_{ions}=\frac{N_{ions}}{N_{A}}\\ m_{C-S-H}=n_{C-S-H}\times M_{C-S-H}=\frac{N_{C-S-H}}{N_{A}}\times M_{C-S-H} \end{array}$$

The adsorption capacity is calculated by Formula (2), which represents the amount of ionic substance adsorbed by the C-S-H gel per unit mass (mol/g), N_ions_ is the number of adsorbed ions, N_A_ is the Avogadro constant (6.02 × 10^23^), N_C-S-H_ is the number of atoms in the C-S-H gel, and C-S-H is the relative atomic mass of each atom. By combining the three formulas, we get:

(3) $$n^{a}=\frac{N_{ions}}{N_{Ca}\times M_{Ca}+N_{Si}\times M_{Si}+N_{O}\times M_{O}+N_{H}\times M_{H}}$$

where N_Ca_, N_Si_, N_O_ and N_H_ are the number of atoms of Ca, Si, O, and H, and M_Ca_, Mi_S,_ and M._O_ M is_H_ are the relative atomic mass of Ca, Si, O, and H. According to Formula (3), the number of sulphate ions increases by 13.26% compared to before the substitution and the number of chloride ions increases by 21.32% compared to before the substitution.

### 3.3. Verification

**Figure 9:**
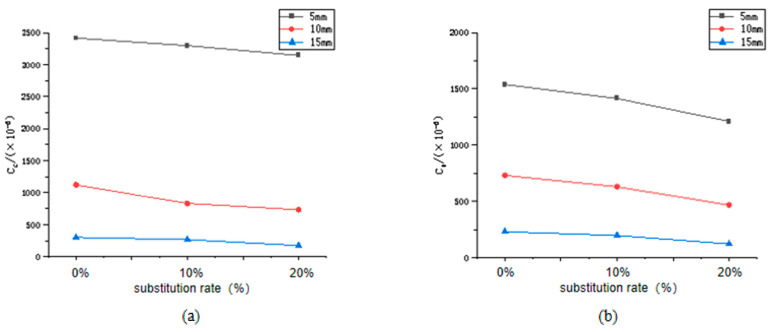
Relationship between the content of chloride and sulphate ions entering and the amount of lithium slag added (**a**) chloride ion content, (**b**) sulphate ion content.

As shown in Figure 9, when the corrosion solution concentration has a 4% chloride ion concentration and a 4% sulphate ion concentration, the contents of the chloride ions and sulphate ions at 5 mm, 10 mm, and 15 mm positions of lithium slag concrete under different lithium slag replacement rates. After the addition of 20% lithium slag, the sulphate ion erosion content and the chloride ion erosion content of the concrete at 5 mm decreased by 13.65% and 15.72%, respectively, and the compressive strength increased by 9.37% after the addition of the lithium slag [26]. After the addition of the lithium slag to the cement, on the one hand, lithium slag has high fineness and volcanic ash activity [27], which has a high specific surface area, reacting with the hydration products of the cement to form a loose and porous gel-like product that improves the compactness of the concrete [28]. Studies have shown that the addition of mineral admixtures can also reduce the directional arrangement of calcium hydroxide crystals [29], strengthening the bonding effect between the interfacial transition zone and the aggregate and slurry [30], reducing the amount of chloride and sulphate ions entering the interior of the concrete through the ITZ [31]. On the other hand, lithium slag is rich in aluminium compounds, such as alumina, which transforms C-S-H, which makes up the largest proportion of cement and plays a crucial role in cement performance, into hydrated calcium aluminosilicate (C-A-S-H) gel [14].This structural change has a critical effect on the performance of cement-based materials, particularly on the physical adsorption process of cement slurry [32]. In order to investigate the mechanism by which changes in the microstructure of concrete affect its physical adsorption, molecular dynamics modelling, and simulation is used in the next section of this article.

Concrete can be considered as a three-phase composite structure: mortar matrix, aggregate, and the interfacial transition zone (ITZ) between matrix and aggregate. Due to the existence of the sidewall effect, the porosity of the slurry near the aggregate surface is higher than that of the matrix part. The migration of water in the forming process may form a water film on the aggregate surface and provide conditions for the migration of Ca^2+^, Al^3+^, and SO_4_^2−^ plasma in the hydration process of the cementitious material. This can lead to the enrichment [33] of CH and AFT near the aggregate surface. Although the volume fraction of the ITZ is smaller than that of the other two-phase components, the ITZ, as the weakest interface in the concrete, has a key influence on the performance of the concrete [34].

**Figure 10:**
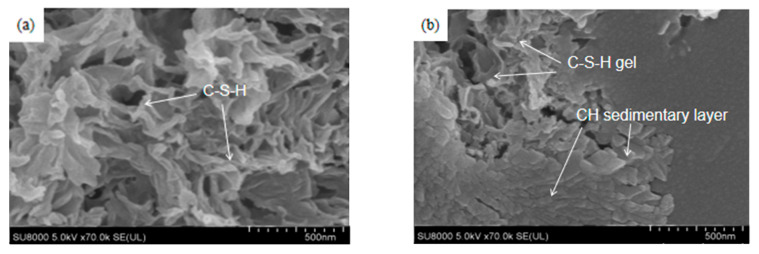

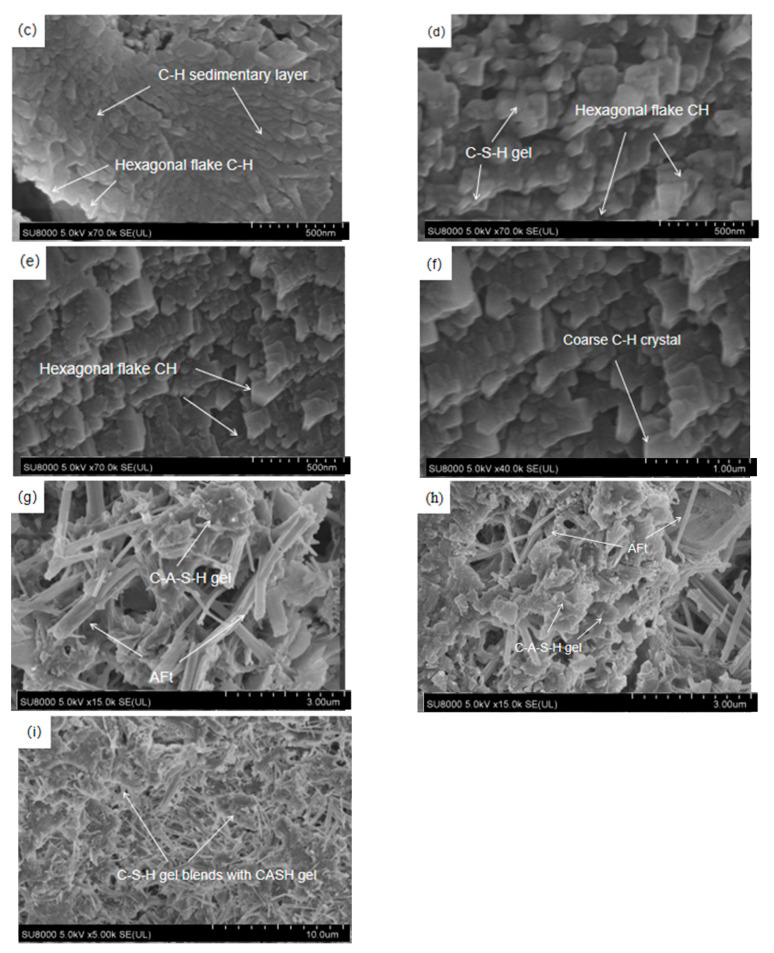
SEM images of the transition zone at the interface with different lithium slag contents of (**a**) 0%Li; 500 nm; C-S-H, (**b**) 0%Li; 500 nm; CH sedimentary layer, (**c**) 0%Li; 500 nm; hexagonal flake CH, (**d**) 10%Li; 500 nm; C-S-H, (**e**) 10%Li; 1 µm; hexagonal flake CH, (**f**) 10%Li; 500 nm; Coarse CH crystal, (**g**) 20%Li; 3 µm; CASH; AFt, (**h**) 20%Li; 3 µm; the connection function of CASH, (**i**) 20%Li; 10 µm; CSH gel blends with CASH gel.

The ITZ is an important factor influencing the adsorption of deleterious ions on concrete. Previous studies have shown [35,36,37,38,39,40,41] that the addition of mineral admixtures with different physical and chemical properties can effectively improve the microstructure and reduce the influence of the ITZ on the transport performance of concrete. In order to study the influence of the lithium slag with a high aluminium admixture on the interfacial transition zone, the 150 × 150 × 150 mm test block was cured according to the standard, cut by tools, and the smooth interface separating the aggregate and mortar was sieved as a specimen, then the specimen was observed using SEM technology. Figure 10 shows the SEM images of the microstructure of the interfacial transition zone with a lithium slag content of 0%, 10%, and 20% in different regions.

In the absence of lithium slag, the microscopic morphology of the interfacial transition zone consists primarily of clustered C-S-H gels as shown in Figure 10a. However, as can be seen in Figure 10b, there are areas in the interfacial transition zone which appear as layered C-H plate crystals. With the addition of 10% lithium slag, no layered C-H sheet crystals could be detected in the interfacial transition zone. Figure 10c shows that the C-S-H gel and some C-H crystals were predominantly mixed in the interfacial transition zone. Although the C-H crystals have an orientation and apparent hexagonal crystal structure, as shown in Figure 10d, unlike the situation without the addition of lithium slag, the C-H crystals are not bonded into sheets and are staggered with each other. The incorporation of lithium slag results in a reduction in the preferred orientation of the C-H crystals, an increase in the contact area between the mortar and the aggregate, and an increase in the bonding strength between the two materials. The microstructure of the interfacial transition zone with a lithium slag content of 20%, as shown in Figure 10e,f, shows no significant C-H crystals at the interface. Mainly rod-shaped Aft crystals and cluster and grass-like C-A-S-H gels are visible. The interfacial C-A-S-H and C-S-H flocculants intertwine to form a network structure, indicating that the addition of lithium slag can modify the microstructure of cement-based materials. Figure 10g shows that some C-A-S-H is bonded to Aft, and it can be observed from Figure 10h that the crystal size of Aft decreases significantly after the addition of mineral admixtures. The incorporation of lithium slag leads to a reduction in the yield and orientation of calcium hydroxide crystals, together with an improvement in the physical adsorption capacity of the sulphate ions and an increase in the content of the sulphate ions collected on the weak surface. The inclusion of lithium slag affects not only the physical adsorption but also the chemical adsorption, resulting in a complex effect on the Al substitution phenomenon of the cement microstructure. In particular, the formation of ettringite becomes more pronounced due to the accumulation of sulphate ions. This shows that the introduction of lithium slag alters the chemical reactions leading to the formation of ettringite and other chemical products, involving the phenomenon of ion concentration.

## 4. Conclusions

(1) Based on the simulation results obtained, it was found that the C-A-S-H model demonstrated physical adsorption of SO_4_^2−^ and Cl^−^. Upon substituting Al, Ca^2+^ was adsorbed on the C-A-S-H model surface due to the charge compensation effect. This results in an increased zeta potential of the model surface, leading to the formation of a double layer structure that enhances the long-range effect and expands the range of action. This indicates that the peak of the van der Waals effect shifts to the right, ultimately increasing the number of combined SO_4_^2−^ and Cl^−^ ions.
(2) Prior to Al substitution, the coordination number of the calcium ions was estimated to be approximately 4. Conversely, after Al substitution, the coordination number of Ca^2+^ ion increased to approximately 7. This phenomenon can be attributed to the increased adsorption of Ca^2+^ ions onto the surface of the C-A-S-H in the model system, leading to an increase in the stern potential. The application of the isothermal adsorption formula revealed a 13.26% increase in the calculated number of sulphate ions compared to pre-Al substitution, while the number of chloride ions was observed to be 21.32% higher than its pre-Al substitution state.
(3) Observation of the interfacial transition zone of concrete was conducted before and after the addition of a high alumina phase admixture at the microscopic level. The results revealed that the interfacial transition zone’s microscopic morphology was primarily comprised of clustered C-S-H gel in the absence of the high alumina phase admixture. Conversely, upon the addition of the high alumina phase admixture, there was a transformation in the microscopic morphology of the interfacial transition zone to the flocculent C-A-S-H and C-S-H. These intermingling entities formed a network structure, improving the stability of the structure and the microstructure of the hydration products.

## Figures and Tables

**Table 1:** Chemical composition of lithium slag (%).

SiO_2_	Li_2_O	MgO	K_2_O	CaO	SO_3_	Na_2_O	Fe_2_O_3_	Al_2_O_3_
54.39	0.77	0.24	0.14	7.98	8.30	0.26	1.40	19.83

**Table 2:** Concrete mix ratio.

Test Block	Lithium Slag	Cement	Gravel	Sand	Water	Water Reducing Agent
Type	(%)	(kg/m^3^)	(kg/m^3^)	(kg/m^3^)	(kg/m^3^)	(kg/m^3^)
PC	0	313.2	1360	657	118.1	2.6
PLiC	10	282	1360	657	118.1	2.6
PLiC	20	250.8	1360	657	118.1	2.6
